# An Unusual Mixed Thyroid Carcinoma: A Surgical and Pathological Rarity

**DOI:** 10.7759/cureus.58291

**Published:** 2024-04-15

**Authors:** Karthikeyan Selvaraj, Aiswerya Shankar, Thanka Johnson, Preethi M

**Affiliations:** 1 General Surgery, Sree Balaji Medical College and Hospital, Chennai, IND; 2 Pathology, Sree Balaji Medical College and Hospital, Chennai, IND

**Keywords:** thyroid carcinoma presentation, turin’s criteria, poorly differentiated thyroid carcinoma, insular carcinoma, mixed thyroid carcinoma

## Abstract

The incidence of mixed thyroid carcinoma of poorly differentiated thyroid carcinoma (PDTC) and papillary carcinoma thyroid is very unusual. PDTC exhibits a high degree of dedifferentiation and histopathological confirmation is done based on Turin’s criteria. This type of carcinoma has a poor prognosis and the survival rates at five and ten years post-diagnosis are significantly lower compared to well-differentiated thyroid carcinomas. Surgery is the best mode of treatment at present. This is a case of a 71-year-old female who underwent total thyroidectomy with modified radical neck dissection which yielded a histopathological variant comprising PDTC and papillary thyroid carcinoma. The patient was followed up with a serial thyroglobulin antibody test and ultrasound of the neck at six months and one year, and both were found to be normal.

## Introduction

Poorly differentiated thyroid carcinoma (PDTC) is an unfamiliar and aggressive type of thyroid cancer. Of all the thyroid cancers, PDTC constitutes a small sector of roughly four to seven percent [1]. PDTC is associated with a lower survival rate and a higher likelihood of recurrence compared to other types of thyroid carcinoma. PDTC is most common in the middle-aged group and slightly older population but the median age it affects is 59 years [2]. It has a 1.6 times greater incidence in women as compared to men [2]. It occurs concomitant with RAS mutation. The most common presentation is a rapidly growing swelling in the neck, hoarseness of voice, difficulty swallowing or breathing, and persistent cough. Additionally, they may exhibit symptoms such as palpitation, excessive sweating, tremors, loss of weight in spite of good appetite, and increased bowel movements. About 70% of patients present with extrathyroidal extension [2]. For this form of thyroid cancer, the most common site of metastasis is primarily to the bone followed by lung and lymph nodes. Although CT scans of the neck and fine needle aspiration cytology (FNAC) are vital investigations, histopathology of the excised tumor is crucial for the diagnosis of PDTC. Turin's criteria form the basis of pathological diagnosis for PDTC. This is a rare case of mixed thyroid carcinoma comprising PDTC and papillary thyroid carcinoma. This case report shows the significance of early detection and surgical treatment of PDTC. It also shows the need for further research into other treatment modalities for PDTC.

## Case presentation

**Figure 1:**
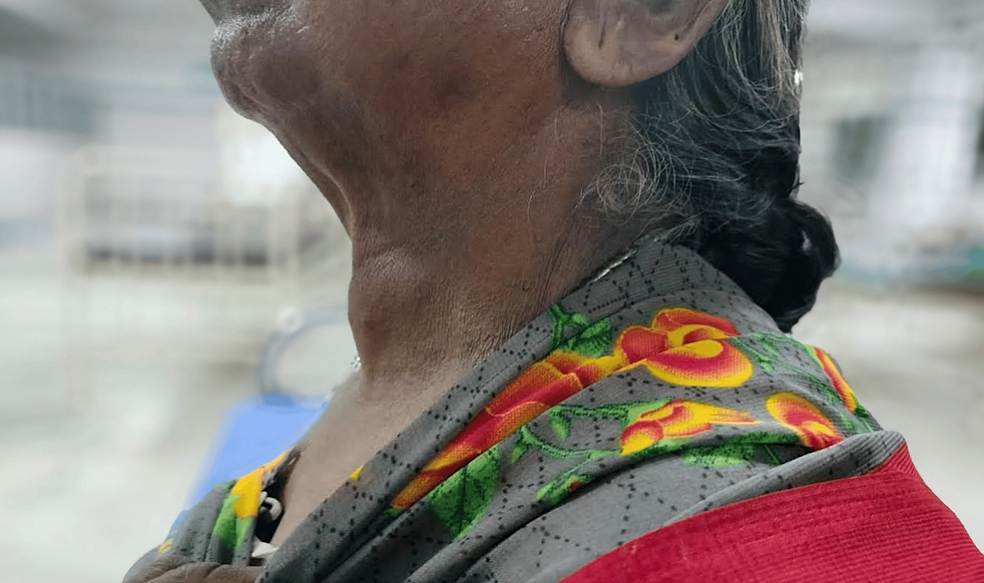
Swelling in front of the neck (left lateral view)

**Figure 2:**
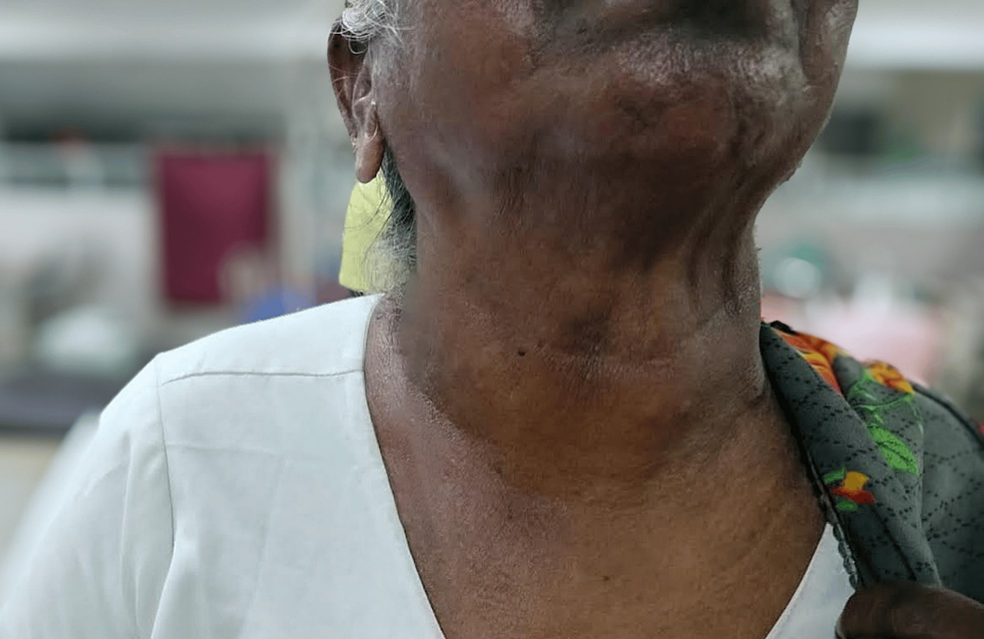
Swelling in front of the neck

In July 2023, a 71-year-old hypertensive female was admitted to the general surgery department at Sree Balaji Medical College Hospital, Chennai, India. She presented with swelling in front of the neck for the past two years. Although initially gradually progressive, there was a sudden increase in the size of the swelling over the past year (Figures 1, 2). She had a history of difficulty swallowing and difficulty in breathing in a lying down position. There was history of change in voice. There was no history of pain, fever. No history suggestive of hypothyroidism or hyperthyroidism; no history suggestive of tuberculosis or distant metastasis

**Figure 3:**
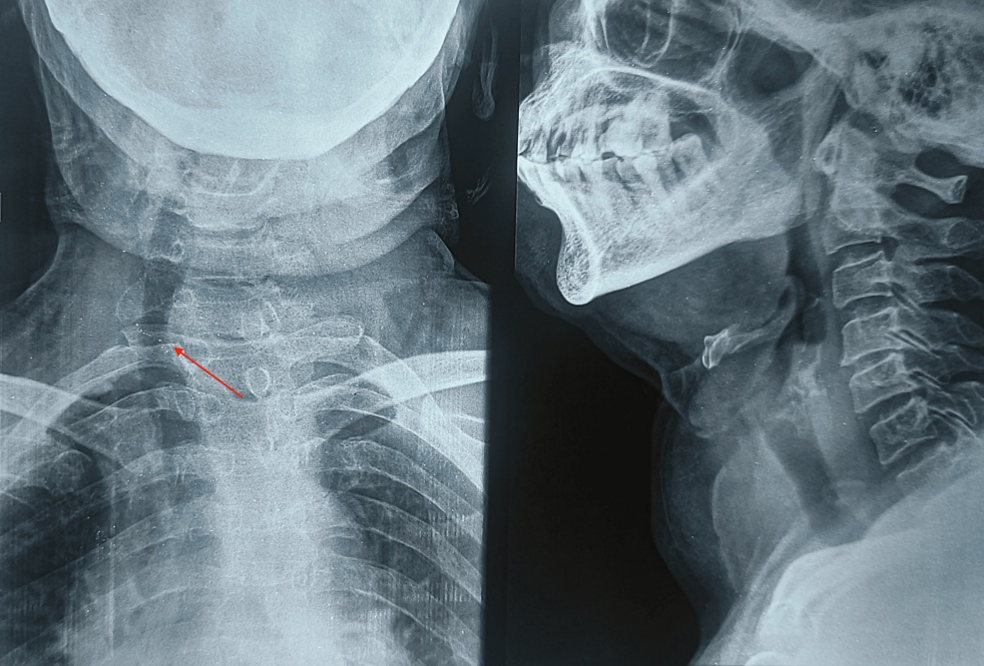
X-ray of the neck showing deviation of trachea towards right side

There was no history of loss of weight or loss of appetite. She had a leukocyte count of 7.6 x 10^9^/L (normal range of 4.0-10 x10^9^/L) and hemoglobin of 10.2g/dL (normal range of 12-16g/dL). Her thyroid function tests showed free triiodothyronine (T3) of 2.83 pg/ml (normal range of 2.1-4.4 pg/mL), free thyroxine (T4) of 0.97 ng/dL (normal range of 0.9-2.4 ng/dL), thyroid-stimulating hormone (TSH) of 4.098 mU/L(Normal range of 0.5-5.0 mU/L), preoperative serum calcium of 9.7mg/dl (8.5 to 10.5 mg/dl), the thyroglobulin antibody level of 6.1 ng/mL (normal range of 0.5-43.0 ng/mL) and thyroid microsomal antibody of 26.19 IU/mL (normal range of 0 to 34 IU/mL). On general examination, there was mild pallor, she was hemodynamically stable. On local examination, there was a 6 x 4 cm solitary swelling in front of the neck, the left side of the midline, ovoid in shape; smooth surface with well-defined borders. Swelling extended from 1 cm below the thyroid cartilage to 1 cm above the suprasternal notch; laterally extended till 2 cm in front of the anterior border of the sternocleidomastoid muscle and medially did not cross the midline. It was variable in consistency, hard in the anterior aspect, and cystic in the lateral aspect. Skin over the swelling appeared to be normal. The swelling moved with deglutition. The lower border of the swelling was visible, there was no evidence of retrosternal extension and no visible pulsations/engorged veins. The trachea was pushed to the opposite side. Bilateral carotid pulsation was equally felt. A solitary enlarged lymph node was present in the level V region, unilateral only on the left side of the neck. It was 20 x 25 mm in size, hard in consistency fixed to the underlying tissue. No eye signs were present. X-ray of the neck showed tracheal deviation to the right side (Figure 3).

**Figure 4:**
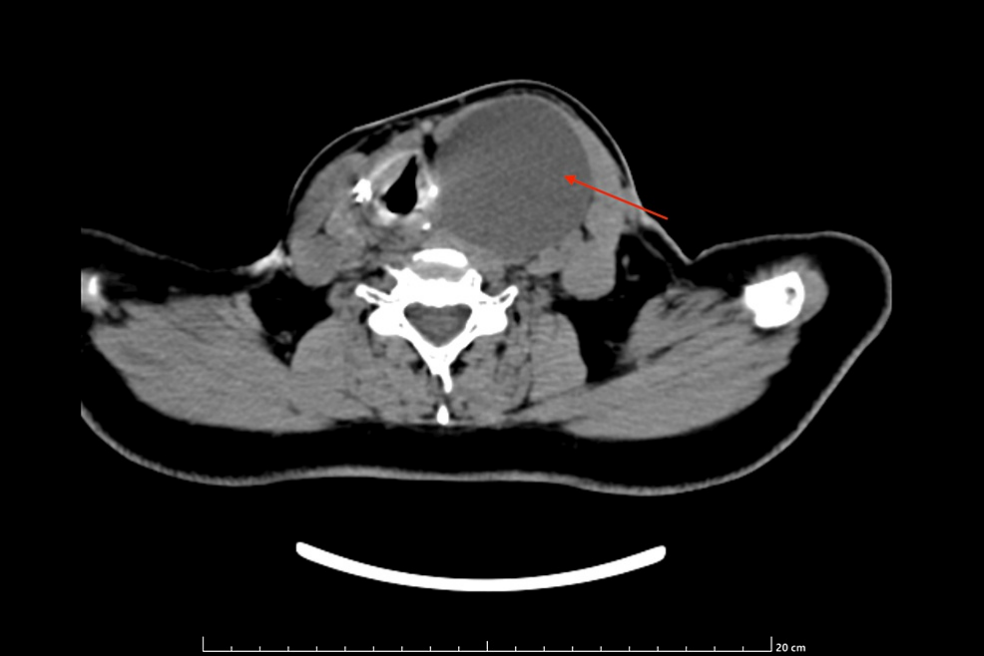
CECT of the neck showing a large, well-defined, lobulated, heterogeneously-enhancing solid cystic lesion in the left lobe of thyroid CECT: Contrast-enhanced computed tomography

**Figure 5:**
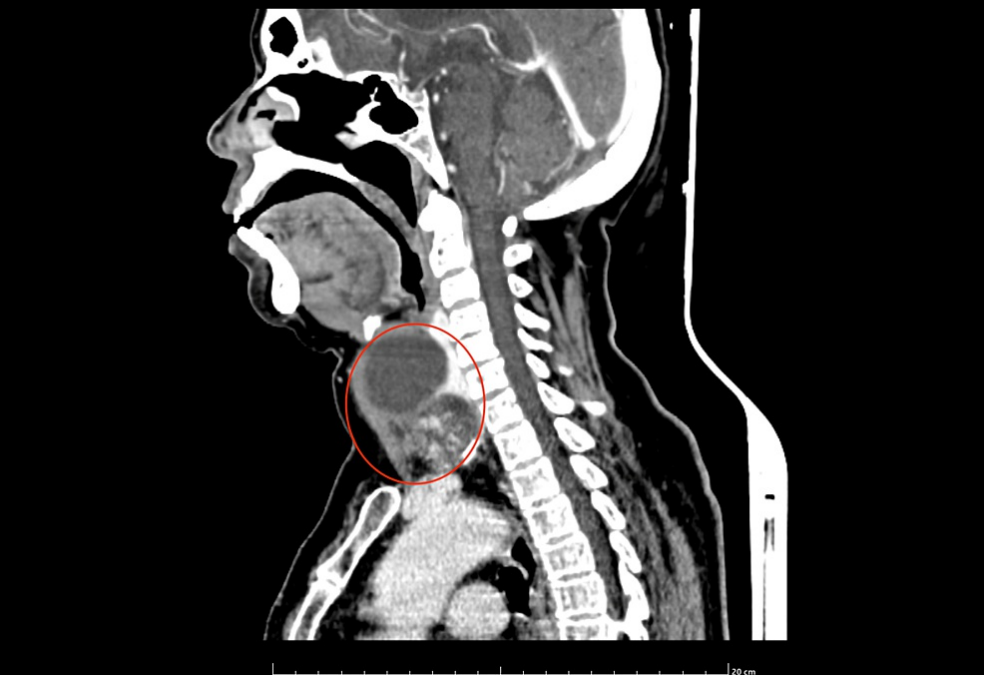
CECT of the neck showing lesion with a rim of calcification CECT: Contrast-enhanced computed tomography

A contrast-enhanced computed tomography (CECT) of the neck showed a large well-defined lobulated heterogeneously enhancing solid cystic lesion in the left lobe of the thyroid. The solid part in the anteroinferior part of the lesion measures ~ 37 x 47 x 30 mm and the cystic part is noted in the posterosuperior aspect of the lesion and measures ~ 56 x 54 x 62 mm. Lesion causing a mass effect in the form of anterior lateral displacement left sternocleidomastoid (SCM), medial displacement of rest of the thyroid gland and trachea towards the right side. Posteriorly, the lesion was seen indenting over the internal jugular vein (IJV) (Figures 4, 5).

**Figure 6:**
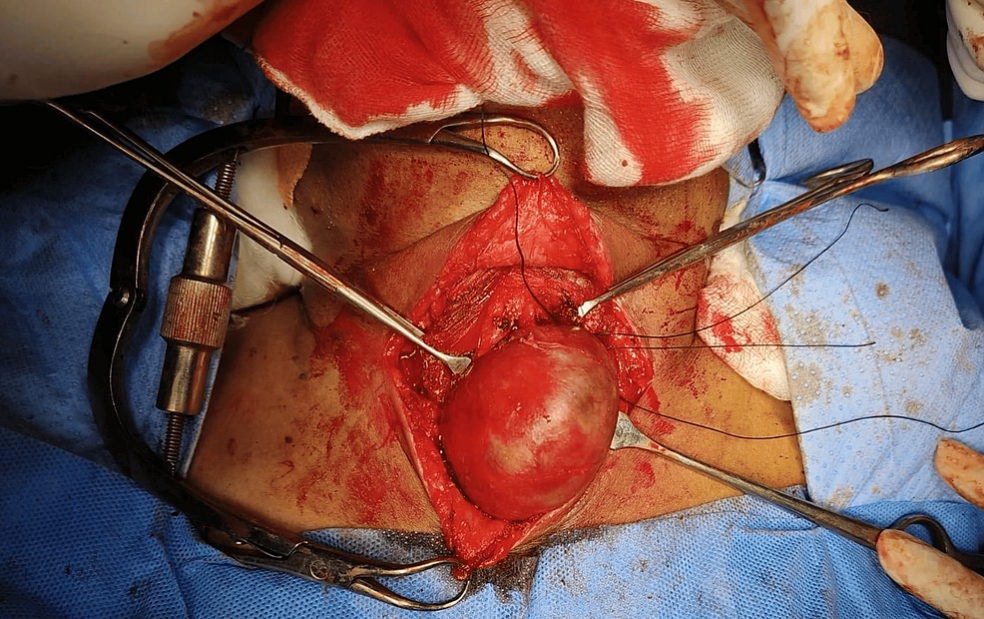
Intraoperative picture of the swelling adherent to the esophagus

Ultrasound-guided FNAC of the unilateral, left level V lymph node smear showed follicular epithelial cells arranged in a papillary pattern and loose clusters in a hemorrhagic background; individual cells showed mild nuclear enlargement with some cells showing nuclear grooving and pseudo inclusions, a picture in favor of papillary neoplasm. After obtaining opinions from surgical oncology, cardiology, and anesthesia, the patient was posted for total thyroidectomy with left modified radical neck dissection. A transverse incision was made 2 cm above the suprasternal notch. Sub-platysmal plane was created. Upper flap raised up to thyroid cartilage, lower flap upto sternoclavicular joint. The left lobe of the thyroid and the tumor were found adherent to the esophagus, pushing the trachea to the right side (Figure 6).

**Figure 7:**
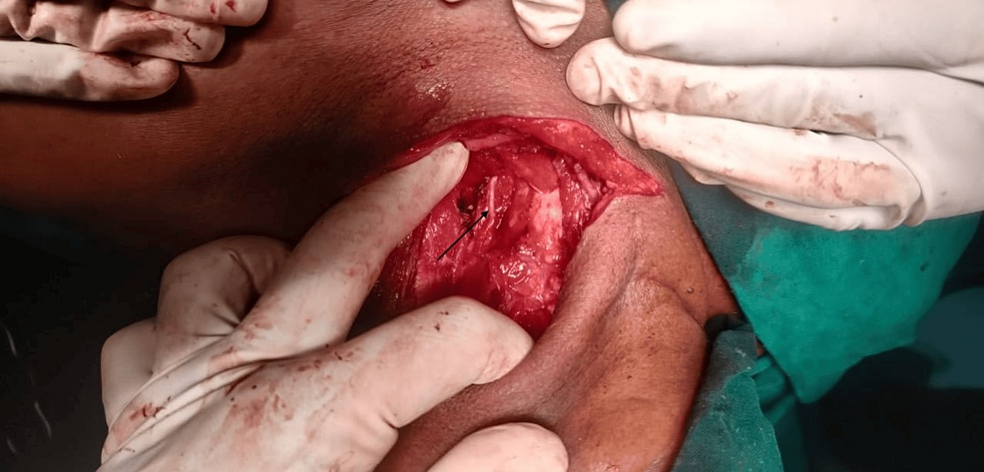
Preservation of left recurrent laryngeal nerve after tumor excision

**Figure 8:**
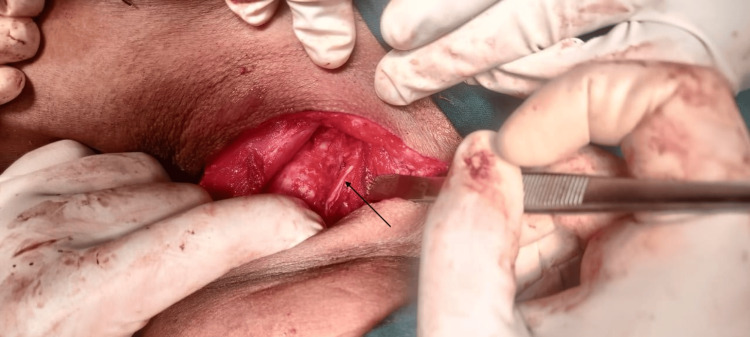
Preservation of right recurrent laryngeal nerve after tumor excision

The bilateral recurrent laryngeal nerve was preserved (Figures 7, 8), and the left parathyroid was removed. The swelling was removed in toto. The neck incision was extended to the left side, flaps raised superiorly to the mandible, and inferiorly to the superior border of the clavicle. Laterally, flaps were raised to the anterior border of the trapezius and medially to the lateral border of the left sternocleidomastoid (SCM). The left spinal accessory nerve was sacrificed. Left IJV and left SCM were preserved. A total of 11 nodes including all nodes over the posterior triangle and level 6 nodes were removed. The tumor along with 11 lymph nodes was sent as a specimen for histopathological examination.

**Figure 9:**
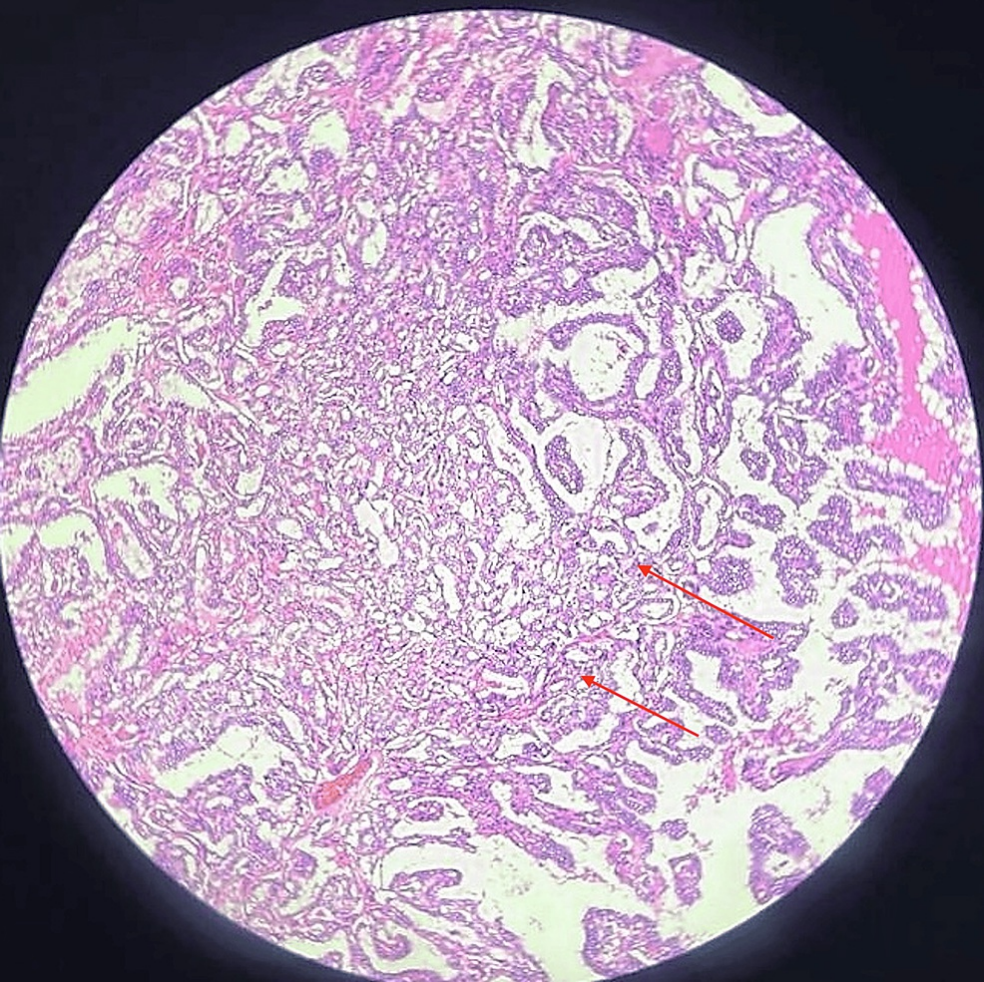
Histopathological image (10x magnification with hematoxylin and eosin stain) showing branching papillae (20%)

**Figure 10:**
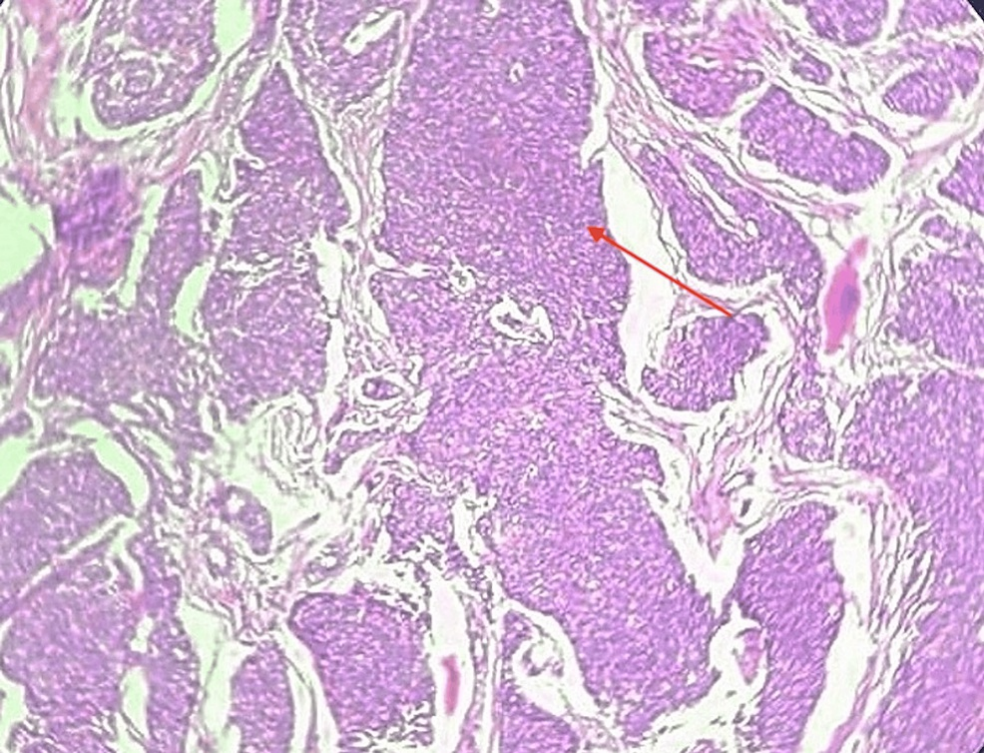
Histopathological image (10x magnification with hematoxylin and eosin stain) showing a solid pattern of tumor arranged in sheets

On histopathological examination, 20% showed a papillary pattern, that is, numerous branching papillae with large ground glass nuclei with overlapping quality with nuclear pseudo-inclusions and nuclear grooves (Figure 9), with a solid pattern of tumor arranged in sheets (Figure 10).

**Figure 11:**
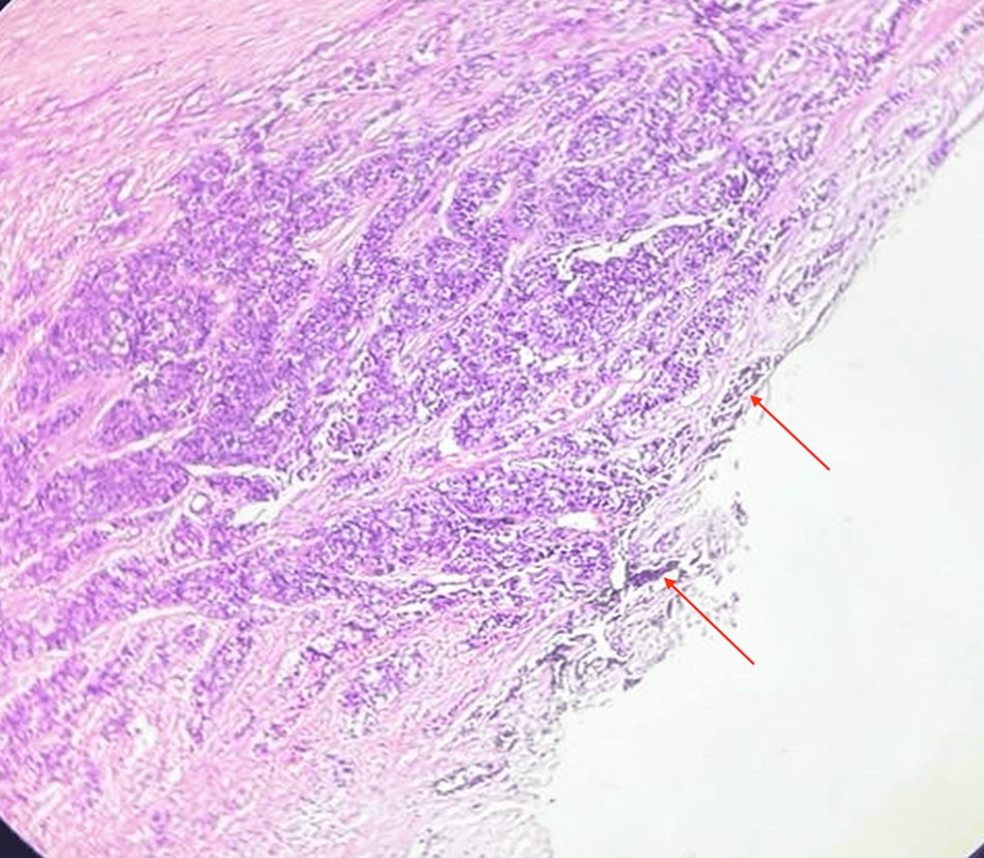
Histopathological image (10x magnification with hematoxylin and eosin stain) showing a solid pattern of tumor arranged in sheets involving the margin seen with India ink

**Figure 12:**
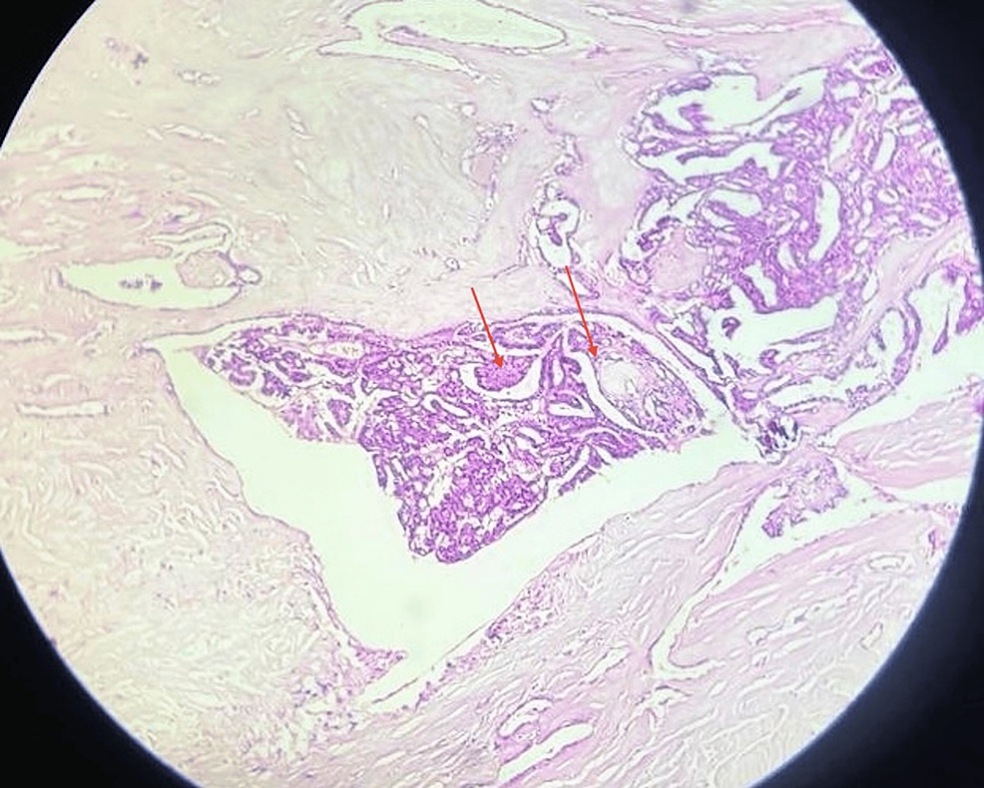
Histopathological image (10x magnification with hematoxylin and eosin stain) showing angioinvasion

The tumor proliferative activity showed a mitotic rate of 8/10 high power field (HPF). Margins were focally involved by poorly differentiated carcinoma (Figure 11). There was 40% tumor necrosis with angioinvasion present in more than four vessels (Figure 12). Lymphatic invasion was present. Microscopic extrathyroidal extension with invasion of the subcutaneous tissue was seen. The tumor was staged as T3bN1a PDTC, 80% with areas of papillary thyroid carcinoma, and 20% with extensive lymphovascular invasion. The absence of clear-cut foci of keratinization, spindle, and giant cells as well as the absence of amyloid deposition ruled out anaplastic carcinoma and medullary carcinoma respectively.

On the second postoperative day, her serum calcium level was 8.9 mg/dl. On a follow-up visit one week post-procedure, the patient was stable with no complaints and the surgical site was healthy. The patient was followed up at the end of the first month, the sixth month, and one year post-procedure. Thyroglobulin antibody was serially monitored every follow-up visit along with an ultrasound of the neck in the sixth month and one year visit and both were found to be normal.

## Discussion

The thyroid tumor for this patient had a combination of PDTC, 80% with areas of papillary thyroid carcinoma, and 20% 20% with extensive lymphovascular invasion. PDTC is an intrusive and unusual thyroid cancer characterized by an intermediate differentiation state. It shows limited follicular cell differentiation. It is neither well differentiated nor undifferentiated and can be classified between the two mentioned types. This poses challenges for accurate diagnosis and effective treatment. PDTC accounts for less than 10% of thyroid carcinomas [1]. It predominantly affects the middle-aged and the elderly. Genetic mutations, specifically alterations in the tumor suppressor genes p53 and p16, lead to the dysregulation of growing cells in turn causing a mutated division. This leads to the progression of poor differentiation of which approximately 15% is caused by BRAF mutation. Mutations of the RAS gene are the most common and are seen in roughly 44% of cases. Age (>45 years) [3], T4a pathological stage [2], extrathyroidal extension [4,5], mitosis and necrosis [3], and distant metastasis at presentation [2] are poor factors of prognosis of this disease. It is often locally advanced with extrathyroidal extension in more than 50% of cases. Nodal, pulmonary, and bone metastases are common. Of the patients, 85% develop distant metastases [6]. Recurrence or metastasis occurs in 60% of cases after treatment. Patients with PDTC have a 62-85% chance of a five-year overall survival rate [2,3]. They have a disease-specific survival rate of 66% [2,7].

For the diagnosis of PDTC, there is no specific immunohistochemical marker as yet [8,9]. PDTC is diagnosed based on the Turin criteria. The Turin consensus defines PDTC as (i) solid/trabecular/insular pattern of growth, (ii) absence of conventional nuclear features of papillary carcinoma, and (iii) showing at least one of the following features, that is, convoluted nuclei, mitotic activity ≥3/10 HPF, or tumor necrosis [10]. Papillary carcinoma on the other hand accounts for about 80% of all thyroid carcinomas. It occurs twice more often in women than in men with a mean age of 30-40 years. Patients are generally euthyroid with a slow-growing painless mass in the neck. Lymph node metastasis is common and most often distant metastasis is to the lungs, liver, and brain. The various differential diagnoses of this swelling are Colloid goiter, calcified multinodular goiter, lymphoma, and thyroid carcinoma from ectopic thyroid tissue in a thyroglossal cyst [11].

PDTC shows very little response to conventional therapies. Radioactive iodine (RAI) and external beam radiation therapy (EBRT) can be tried to target residual disease or distant metastases. Although it has not shown much evidence of improvement in survival [12], it can be useful in patients with neck node involvement [1]. According to a study done in Saudi Arabia, the most common neck node involved is level IV followed by level III nodes [13]. In the above case, level V lymph nodes were enlarged which makes it rare. The main treatment modality still remains surgical, comprising modified radical neck dissection with total thyroidectomy. The prognosis for this type of carcinoma is poor, as there is an increased possibility of local and distal metastasis. Although chemotherapeutic agents such as sorafenib and lenvatinib are FDA-approved, there is very little evidence to show their effectiveness in the treatment of poorly differentiated thyroid cancers [14]. Recurrence rates and follow-up care are crucial aspects that contribute to the prognosis. For follow-up, close surveillance enables prompt intervention and treatment adjustment, ultimately improving patient outcomes and prognosis in individuals with poorly differentiated carcinoma of the thyroid.

## Conclusions

This patient was diagnosed with mixed thyroid carcinoma involving poorly differentiated thyroid carcinoma (PDTC) which is an aggressive form of cancer that carries a high mortality rate. Early detection and appropriate surgical treatment were extremely crucial in improving her chances of survival. Mixed thyroid carcinoma poses significant challenges in treatment and surveillance. In metastatic cases and radioiodine refractory cases, there is a lack of effective treatment options as chemotherapy has proven to be only minimally effective. Such poor prognosis associated with this malignancy underscores the need for further research and clinical trials to improve outcomes for patients diagnosed with mixed thyroid carcinomas involving PDTC.
